# Current Evidence in Robotic Colorectal Surgery

**DOI:** 10.3390/cancers17152503

**Published:** 2025-07-29

**Authors:** Franziska Willis, Anca-Laura Amati, Martin Reichert, Andreas Hecker, Tim O. Vilz, Jörg C. Kalff, Stefan Willis, Maria A. Kröplin

**Affiliations:** 1 Department of General, Visceral, Thoracic, and Transplant Surgery, University Hospital Giessen, 35392 Giessen, Germany; franziska.willis@chiru.med.uni-giessen.de (F.W.);; 2 Department of General, Visceral, Thorax and Vascular Surgery, University Hospital Bonn, 53127 Bonn, Germany; 3 Department of General, Visceral, and Thoracic Surgery, Klinikum der Stadt Ludwigshafen am Rhein, 67063 Ludwigshafen, Germany; 4 Chirurgische Klinik A, Klinik für Allgemein-, Viszeral-und Thoraxchirurgie, Klinikum der Stadt Ludwigshafen am Rhein, 67063 Ludwigshafen, Germany

**Keywords:** colorectal cancer, minimally invasive surgery, robotic surgery, laparoscopic surgery

## Abstract

Colorectal surgery has undergone significant advances over the past few decades, driven by the evolution of minimally invasive techniques, particularly laparoscopy and robotics. While laparoscopy is widely recognized for its short-term benefits and oncological safety, the increasing adoption of robot-assisted surgery (RAS) has generated considerable debate regarding its clinical benefits, economic implications, and overall impact on patient outcomes. This narrative review synthesizes the existing evidence, highlighting the clinical and economic aspects of RAS in colorectal surgery, while exploring areas for future research. The findings suggest that RAS offers potential technical advantages, including increased precision, three-dimensional visualization, and improved ergonomics, particularly in anatomically complex scenarios such as low rectal resections. Still, its superiority over laparoscopy remains inconclusive and current evidence is mixed. For colon cancer, meta-analyses and analyses of large cohorts suggest lower conversion rates and faster recovery with RAS, although data are mostly retrospective and lack long-term oncological endpoints. In rectal cancer, emerging evidence from randomized controlled trials demonstrates improved short-term outcomes. Additionally, the recently published three-year results of the REAL trial are the first to demonstrate enhanced oncological outcomes following RAS. However, findings remain inconsistent due to methodological heterogeneity, the absence of patient stratification, and limited data on long-term survival and cost-effectiveness. The available evidence indicates that RAS may offer advantages in selected patient populations, particularly for anatomically complex procedures. Yet, its overall utility remains uncertain. Future studies should emphasize high-quality randomized trials, stratified subgroup analyses, and standardized economic evaluations to better define the role of RAS in colorectal surgery.

## Simple Summary

Minimally invasive techniques like laparoscopic and robotic surgery have transformed the treatment of colon and rectal cancer. While robotic systems are increasingly used and offer technical advantages such as better precision and visibility, it is still unclear whether they lead to better outcomes for patients compared with standard laparoscopy. This review looks at the current evidence comparing both approaches, focusing on short- and long-term patient outcomes, surgical challenges, and economic factors. The goal is to understand where robotic surgery truly adds value, especially in complex cases such as tumors in difficult anatomical locations. Our findings suggest that robotic surgery may offer benefits in certain situations, but more high-quality studies are needed to confirm this and to assess long-term results and cost-effectiveness. This research helps to identify where future studies should focus and supports informed decision-making in surgical practice.

## 1. Introduction

The adoption of minimally invasive surgery (MIS) in colorectal resections began in the early 1990s with the first laparoscopic colectomy (LC) performed by Jacobs et al. [1]. Initially, concerns regarding oncological safety limited broader uptake. However, subsequent randomized trials—most notably the COST [2], COLOR [3], and CLASICC [4] studies—demonstrated oncological equivalence to open surgery, thereby establishing laparoscopic colectomy as a standard approach. Based on these developments, MIS has become a central component of colorectal surgical practice and is associated with multiple perioperative benefits: it has been shown to lead to a significant reduction in access-related trauma, resulting in decreased postoperative pain, accelerated mobilization, reduced wound infections, and enhanced gastrointestinal motility [5,6,7]. Numerous studies have confirmed that MIS is associated with reduced rates of postoperative complications, decreased length of hospital stays, and improved cosmetic satisfaction [5,6,7]. Despite these advantages, conventional laparoscopy presents challenges, including a steep learning curve, limited instrument mobility due to rigid, non-articulating tools, and unnatural hand–eye coordination from the absence of three-dimensional vision [7,8,9].

**Figure 1:**
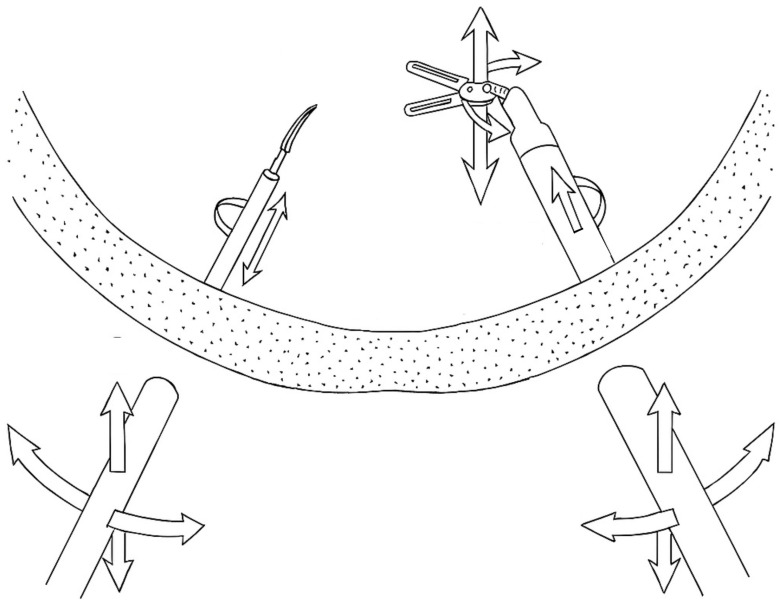
Comparison of conventional laparoscopic and robotic surgical instrument mechanics. This schematic illustrates the fundamental mechanical differences between conventional laparoscopic and robotic surgical instruments. (**Left Panel**): Conventional laparoscopic instrument. A rigid, straight-shafted laparoscopic instrument is inserted through the abdominal wall, which acts as an invariant point. Movement of the instrument within the patient’s body is restricted by this pivot point, offering only 4 degrees of freedom. These include in-and-out translation along the shaft, rotation around the shaft’s axis, and two degrees of angular movement (pitch and yaw) resulting from the pivoting action. The instrument tip lacks intrinsic articulation, meaning that its movement is entirely dependent on the manipulation of the handle and the fulcrum effect. (**Right Panel**): Robotically driven instrument. A robotic surgical instrument, featuring a complex, articulated “wristed” tip, is inserted through an abdominal wall entry point. Unlike the conventional instrument, the robotic instrument’s internal articulation allows its tip to move independently of the external pivot point. This design enables highly precise and intuitive movements (e.g., rotation, flexion, and extension) that mimic the dexterity of a human hand and wrist within the confined surgical space. The combined movement of the robotic arm and the instrument’s internal articulation typically provides 7 degrees of freedom at the instrument tip, significantly enhancing maneuverability and precision compared with conventional laparoscopic approaches. Illustration was generated using OpenAI’s ChatGPT (GPT-4, July 2025 version) with image-generation capabilities.

**Figure 2:**
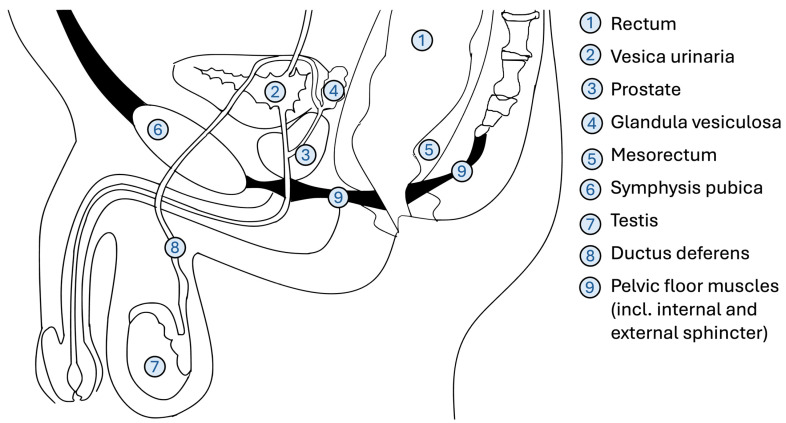
Anatomical topography of pelvic organs. Schematic sagittal section of pelvic organs in the male pelvis, demonstrating the close spatial relationships within the small pelvis and highlighting the anatomical proximity between the rectum, bladder, and prostate. This image was generated using OpenAI’s ChatGPT (GPT-4, July 2025 version) with integrated image-generation functionality.

To overcome key limitations of conventional laparoscopy—such as restricted instrument mobility, unstable camera control, and ergonomic strain—robotic surgical systems were developed. Robot-assisted surgery (RAS) addresses many of these limitations. The da Vinci system received FDA approval in 2000 [7], offering enhanced articulation with tremor suppression and greater degrees of freedom through articulated tools [7,10] (see Figure 1). Additionally the system offers 3D visualization, and greater precision in confined anatomical spaces such as the pelvis [7]. Since 2017, several additional robotic platforms have obtained FDA approval, triggering a rapid expansion of robotic applications and spurring the development of novel features such as haptic feedback, eye-tracking, and modular control systems [11,12]. These technological advances have fueled growing interest in robot-assisted colorectal surgery, particularly in anatomically complex procedures, with the potential to reduce intraoperative complications and conversion rates to open surgery, and to enhance both short- and long-term outcomes [7,10]. RAS appears to be particularly advantageous for complex procedures in confined spaces, such as the pelvic cavity [7,10] (see Figure 2). In these instances, robotic systems facilitate the precise identification and meticulous dissection of structures such as the inferior hypogastric plexus, ureters, and gonadal vessels, thereby reducing the likelihood of postoperative functional disorders affecting bowel or sexual function [7,11].

Anatomic variations contribute to the considerable variability in the complexity of colorectal resections. Laparoscopic sigmoid resection and right hemicolectomy are relatively simpler and faster to master compared with left hemicolectomy, radical transverse colon resection, or low anterior resection. Intricate dissection around central vessels or within the confined pelvic space is less commonly performed laparoscopically in anatomically challenging situations. However, robotic platforms may offer a more effective solution in these cases. Conversely, RAS is associated with higher financial costs and its extensive implementation is also constrained by economic factors.

This review aims to provide a structured comparison of robot-assisted and laparoscopic colorectal surgery, focusing on short- and long-term clinical outcomes, cost implications, and anatomical subgroups. Particular attention is paid to the distinction between colon and rectal resections and the specific technical challenges each entails.

## 2. Methods

This review is designed as a narrative overview of the current evidence on robotic colorectal surgery. An orienting literature search was conducted in MEDLINE (via PubMed) and the Cochrane Central Register of Controlled Trials (CENTRAL). Search terms included combinations of “robotic surgery”, “laparoscopy”, “colorectal surgery”, “colon resection”, and “rectal cancer”, with a focus on identifying randomized controlled trials, meta-analyses, and systematic reviews. The search strategy was informed in part by a previously published systematic review by the authors on robotic versus laparoscopic rectal resections [13], and was expanded to cover the broader field of colorectal procedures.

All publications deemed relevant to the clinical and anatomical questions addressed in this review were considered and, where appropriate, cited. The search was not based on predefined inclusion or exclusion criteria, and no formal screening process was applied. No formal quality assessment was conducted, as this is a narrative review. However, the methodological limitations of the cited studies are discussed throughout this manuscript where relevant.

## 3. Colon Resections

### 3.1. Benefits of MIS

According to the extant literature, high-quality randomized controlled trials (RCTs) from the early 2000s demonstrated that oncological outcomes, including extent of resection, lymph node harvest, disease-free survival, and overall survival, are comparable between open and laparoscopic colorectal resections [4,14,15]. These findings were further validated by meta-analyses of large patient cohorts. However, laparoscopic surgery consistently demonstrated superior short-term outcomes, including fewer postoperative complications, faster recovery, and shorter hospital stays [15,16,17]. Notably, the oncological safety and recovery benefits of laparoscopy were confirmed in high-risk populations, such as patients with T4 colon cancers [18] and those over 80 years old [19]. Yet, despite its clinical importance, evidence regarding the influence of tumor location remains limited. A single RCT and several single-center studies have identified the advantages of laparoscopy for right hemicolectomy, including improved lymph node harvest, lower recurrence and complication rates, and shorter hospital stays, albeit with longer operative times [20]. For left-sided colectomies, study protocols have been published, but results are pending [21]. For transverse colon cancer, a retrospective study reported comparable outcomes between laparoscopic and open approaches [22]. However, a cumulative meta-analysis of 39 randomized and non-randomized studies involving 5782 patients found that intraoperative conversion from laparoscopic to open surgery was associated with higher complication rates and poorer disease-free survival compared with patients undergoing exclusively open or laparoscopic procedures [17]. Notwithstanding these evidence gaps, LC has evolved into the standard therapeutic approach.

### 3.2. Laparoscopic vs. Robotic Approaches

While the advantages of MIS compared with open surgery are well-established and minimally invasive approaches are recommended for most patients, the comparative evidence between RAS and LC remains heterogeneous and limited by methodological shortcomings. Most of the available studies are retrospective and non-randomized, with varying definitions of outcomes, lack of long-term oncological endpoints, and inconsistent segmental stratification. Additionally, most studies included in meta-analyses are of a retrospective cohort nature, thereby diminishing the significance of the results obtained. These limitations hinder definitive conclusions, despite the increasing body of literature. Table 1 provides an overview of key comparative studies.

Regarding all colon resection, a meta-analysis by Negruț et al. [24], which included over 50,000 patients from 21 studies (only 3 of which were prospective), found that RAS was associated with reduced conversion rates and shorter hospital stays. But, it was also associated with longer operative times and higher costs. However, the study did not stratify outcomes by tumor location, and its conclusions are limited by heterogeneity in surgical technique and patient selection. Moreover, no oncological outcomes were evaluated. In a similar vein, the systematic review by Gonçalves et al. [23], which was based on a mere four randomized trials, was underpowered for the majority of outcomes. The study reported a reduction in length of stay, but an increase in wound complication rates in the RAS group. The limitations of the study are evident in the small sample sizes (fewer than 150 patients per arm) and the low GRADE ratings across all endpoints, which preclude any definitive recommendations.

For right-sided colon cancer, several recent meta-analyses indicate that RAS may offer certain perioperative advantages over LC [25,26,27,29]. A recent meta-analysis by Meyer et al. including 16 studies and over 20,000 patients indicated that RAS is associated with a higher lymph node yield, lower conversion rates, earlier return of bowel function, and shorter hospital stays, albeit at the cost of longer operative times [29]. A previous meta-analysis of 20 studies also demonstrated advantages of robotic surgery regarding complication and anastomotic leakage rates [25]. Corroborating these findings, a meta-analysis by Tschann et al. [26] including 25 studies and over 16,000 patients reported significantly lower conversion rates, reduced intraoperative blood loss, and shorter hospital stays in the robotic group, whereas operative time was found to be significantly prolonged. Similar findings were reported by Zheng et al. [27], who conducted a meta-analysis with a specific focus on right hemicolectomy. Nonetheless, a common limitation across these analyses is their reliance on retrospective data, with a significant proportion of studies failing to adjust for relevant patient- and tumor-specific variables, thereby introducing considerable selection bias. The only published RCT on this topic, involving 35 patients in each group, did not demonstrate superiority of RAS over conventional laparoscopy in terms of complication rates, postoperative pain, hospital stay duration, or tumor-free resection margins. But, operative times were significantly longer in the RAS group, and the associated costs were higher [30].

More than 90% of anastomoses in minimally invasive right hemicolectomy are created extracorporeally via a midline mini-laparotomy, which also serves as the extraction site for the specimen [33]. Advocates of RAS argue that robotic systems facilitate intracorporeal anastomosis, allowing the specimen to be retrieved through a smaller Pfannenstiel incision. This approach has the potential to result in a reduction in postoperative ileus, reduced pain, and the risk of incisional hernias as a long-term complication [43]. A meta-analysis of seven RCTs corroborates these claims, reporting a significantly lower incidence of ileus in RAS. However, no differences were found regarding anastomotic leakage, wound infections, lymph node harvest, perioperative morbidity, or hospital stay duration, while robotic surgery was associated with longer operative times as well [44]. The first results of the MIRCAST study, an international, multicenter, prospective, observational four-cohort study investigating both intra- and extracorporal anastomosis in RAS and LC, indicate lower overall complication rates, and specifically reduced postoperative delayed bowel function and ileus after intracorporal anastomosis. However, the study did not demonstrate significant differences in the primary composite endpoint, defined as the absence of wound infection and any other major complication within 30 days after surgery [45].

The evidence supporting left-sided colectomies is less robust and methodologically weaker. Two propensity-score-matched analyses from the Denmark national cohort [39] and the ACS-NSQIP database [38] describe lower conversion rates and longer operation times. Rein et al. also found that RAS is associated with a higher lymph node yield [39]. The findings of both studies demonstrated that there were no statistically significant differences in terms of morbidity and mortality between the two groups [38,46]. A meta-analysis focusing on left colectomies also describes lower conversion rates for RAS, without significant differences regarding any other outcomes [42].

For transverse colon resections, no high-quality comparative data are available. This segment remains particularly underrepresented in both RCTs and retrospective studies, likely due to its relative rarity and technical complexity. However, a meta-analysis of four retrospective studies considering merely 300 patients reports shorter LOS and longer operations times for RAS as well, while there were no significant differences in conversion rates, time to bowel movement, or morbidity [28]. Given the limited sample size and heterogeneity of the included studies, it is not possible to draw reliable conclusions regarding the comparative efficacy or safety of RAS versus LC in this subset.

While the majority of studies do not differentiate between tumor stage and size, a retrospective study by Kamel et al. using NCDB data focuses on advanced T4b cancers with infiltration of adjacent organs necessitating multivisceral resection [36]. Conversion to open surgery was necessary in more than one-third of patients treated laparoscopically (37%), whereas in RAS, conversion to open surgery was performed in merely 12% of cases. This resulted in a significantly shorter LOS in the RAS group, without significantly affecting 5-year survival. In complex cases, the advantages of the robot, such as the enhanced range of motion and improved 3D visualization, which enable greater precision, may explain the relatively greater difference in conversion rates between RAS and LC.

Regarding long-term oncological outcomes, the evidence is still limited. A meta-analysis of five studies involving 523 patients found no significant differences in disease-free or overall survival between robotic and laparoscopic approaches [47]. The only RCT by Park et al. comparing RAS and LC did not find any differences in disease-free (DFS) and overall survival (OS) [31]. Additionally, OS and DFS were equivalent in large matched registries [34,35]. Despite widespread reporting of reduced conversion-to-open rates with RAS, the clinical significance of this advantage remains uncertain. While multiple comparative studies—especially from national audits and registry data—consistently demonstrate a lower conversion rate with RAS [32], most analyses are limited to short-term endpoints (30–90 days), and very few explore whether this reduction translates into improved long-term oncological outcomes, patient-reported quality of life, or lower rates of incisional hernia, bowel obstruction, or long-term healthcare expenditures [32,33,34,35,36,38]. Notably, large matched cohort studies have shown equivalent five-year OS across RAS and LC groups, despite significant differences in conversion profiles [34,35]. A trend toward improved survival with RAS in high-risk subgroups, such as patients with T4b tumors, has been reported, but failed to reach statistical significance [36]. Thus, the long-term oncologic impact of the robotic conversion advantage remains unresolved and warrants prospective investigation, ideally through randomized trials stratified by conversion risk. Whether RAS confers true long-term oncologic superiority remains an open question. To date, only one RCT includes five-year follow-up, limited to right-sided colectomy [30,31]. Though major registries such as NCDB [34] and the Danish national database [35] report broadly equivalent DFS and OS rates, these findings are not consistently replicated in smaller studies [48], and the observational nature of most data leaves room for unmeasured confounding. Future research should focus on long-term outcomes through RCTs and explore pathologic correlates like mesocolic-plane integrity in addition to lymph node yield and resection margins to uncover subtle quality differences between techniques.

## 4. Rectal Resections

### 4.1. Benefits of MIS

Given the advantages of laparoscopic surgery in short-term outcomes, the proportion of laparoscopic rectal resections has steadily increased over the past decade, currently accounting for 40–50% of cases [7,49,50]. However, the technical limitations of conventional laparoscopy, particularly in confined and narrow spaces such as the male pelvis or in obese patients, can lead to higher conversion rates to open surgery or, in the worst-case scenario, tumor manipulation or perforation [9].

This incites deliberation on the question of whether the presumed short-term benefits of laparoscopy justify the potential for worse oncological outcomes due to a necessary conversion. While the COLOR-II and COREAN trials demonstrated oncological equivalence between laparoscopic and open approaches [5,51], the ACOSOG [52] and ALaCaRT [53] trials yielded different conclusions. The latter studies used a composite endpoint assessing the quality of mesorectal preparation and the tumor-free status of the circumferential and distal resection margins, suggesting that open surgery might outperform laparoscopy in these aspects. However, the validity of the composite endpoint is not well established, and no definitive conclusions regarding hard oncological endpoints can be drawn. Furthermore, long-term data from the ACOSOG trial showed no significant differences in 2-year disease-free survival, local recurrence rates, or distant metastases [54]. A 2014 Cochrane Review, which analyzed 14 RCTs on this topic, concluded that laparoscopic total mesorectal excision (TME) achieved similar long-term survival rates to open TME, while offering better short-term outcomes in cases of non-locally advanced rectal cancer [49]. Another meta-analysis focusing solely on RCTs with data on circumferential resection margins also found comparable oncological quality of the specimens, regardless of the surgical approach [55]. Additionally, recent analyses of data from the Swedish Colorectal Cancer Registry confirmed that laparoscopic surgery for rectal cancer is equivalent to open surgery in terms of tumor resection quality. The analysis also highlighted favorable short-term outcomes for MIS in non-advanced disease [56].

### 4.2. Laparoscopic vs. Robotic Approaches

The anatomical location of the rectum within the pelvis, in close proximity to urogenital organs and with numerous muscles and nerves critical for continence and sexual function, creates significant challenges for conventional laparoscopy (see Figure 2). The enhanced precision offered by robotic systems is proposed to reduce intraoperative complications, reduce conversion rates, and improve both short- and long-term outcomes.

However, so far, the current literature provides no conclusive evidence for the superiority of RAS in rectal cancer. Interestingly, study results vary depending on the design. While database analyses suggest that robotic surgery is associated with lower conversion rates and comparable or slightly shorter postoperative hospital stays, with similar perioperative mortality rates [57,58], cohort studies report a reduction in overall postoperative complications and shorter hospital stays [14,59,60]. Conversely, RCTs provide only modest support for these findings [61,62,63,64].

A meta-analysis by Hoshino et al. investigated the influence of study designs on outcomes, showing that cohort studies specifically indicate reduced postoperative complications and shorter hospital stays after robotic-assisted rectal resection, whereas this effect was not observed in RCTs [65]. Additionally, existing RCTs report conflicting results. For instance, the ROLARR trial (2017) [63] and other studies like that by Tang et al. (2020) [64] found no clear advantage of robotic-assisted rectal resection over laparoscopy, with similar short- and long-term outcomes. Similarly, Park et al. (2023) reported comparable results in terms of the completeness of TME [10]. In contrast, the REAL trial, a multicenter RCT by Feng et al. (2022), demonstrated that RAS resulted in fewer postoperative complications, shorter hospital stays, and improved oncological specimen quality compared with laparoscopy [62]. The recently published 3-year results, however, are the first to demonstrate the superiority of robotic rectal resections [66]. Three-year local recurrence-free survival and disease-free survival were both significantly improved with RAS (locoregional recurrence rate: 1.6% vs. 4.0%, disease-free survival: 87.2% vs. 83.4%). However, these findings have not yet translated into improved overall survival. Furthermore, patients in the robotic group experienced better functional outcomes in terms of urinary function, as well as male and female sexual function [66]. This study had a larger sample size than earlier single-center studies. Additionally, it is important to note that the study only included patients with tumors in the mid and lower rectum, where the technical advantages of robotic systems are said to be most evident. The results of this study have not yet been included in published reviews, and no meta-analyses have conducted subgroup analyses addressing this specific context.

Although these anatomical scenarios are frequently cited as key indications for RAS, robust, subgroup-specific evidence is lacking. Most existing meta-analyses and randomized trials have not performed stratified analyses for high-risk populations, such as male patients with narrow pelvises, obese individuals, and patients with low rectal tumors. Consequently, the presumed advantages of robotic surgery in these contexts remain largely theoretical.

A recent scoping review based on level 1a evidence [13] and an ongoing Cochrane review intend to evaluate these patient subgroups [67]. The list of RCTs comparing robotic versus laparoscopic rectal resection in Table 2 reflects the different results of the studies. Accordingly, meta-analyses of RCTs have yielded divergent results as well [13]. While three studies reported significantly longer operative times for RAS [6,68,69], two studies found no difference [70,71]. Similarly, four meta-analyses identified reduced conversion rates in RAS [6,69,70,71], whereas two studies did not detect any differences [68,72]. Four meta-analyses investigated the time of first bowel movement, and all of them reported reduced time to first bowel movement [6,69,70,71]. However, this effect was only statistically significant in two studies [6,71]. This divergence has been analyzed in a scoping review of level 1a evidence, which identified substantial methodological heterogeneity between meta-analyses, inconsistencies in the included trials, and questionable strategies in managing inter-study variability [13].

In summary, although extant evidence suggests the potential benefits of robot-assisted rectal resection, contradictory findings leave the overall picture inconclusive. This uncertainty is highlighted in a recent scoping review based on Level 1a evidence [71]. A comprehensive Cochrane meta-analysis, incorporating detailed subgroup analyses, is anticipated to offer more precise insights into this matter [73].

**Table 2:** Current evidence comparing robot-assisted and laparoscopic surgery for rectal resections.

RCT	Population		Outcomes				
	Robotic Surgery	Laparoscopic Surgery	Complete Total Mesorectal Excision (TME) (RR + 95% CI)	Conversion to Open Surgery (RR + 95% CI)	Operation Time (Minutes, MD + 95% CI)	Lenth of Hospital Stay (Days, MD + 95% CI)	Local Recurrence (3-Year Follow Up)
Baik et al., 2008 [74]	18	16	1.16 [0.90, 1.51]	0.18 [0.01, 3.47]			
Rodriguez et al., 2011 [75]	28	29		1.04 [0.16, 6.86]	24.30 [5.00, 43.60]	0.10 [−3.79, 3.99]	
Tolstrup et al., 2017 [76]	25	26		0.10 [0.01, 0.75]	−18.00 [−45.64, 9.64]	−0.60 [−4.28, 3.08]	
Jayne et al., 2017 ROLARR Trial [63]	237	234	1.02 [0.91, 1.13]	0.66 [0.38, 1.15]	37.50 [21.91, 53.09]	−0.20 [−1.25, 0.85]	0.65 [0.33, 1.28]
Kim et al., 2018 [77]	66	73	1.03 [0.87, 1.22]	3.31 [0.14, 79.96]	111.40 [86.91, 135.89]	−0.50 [−2.39, 1.39]	
Debakey et al., 2018 [61]	21	24	1.37 [0.96, 1.96]	0.57 [0.06, 5.86]			
Feng et al., 2022, 2025 REAL Trial [62,66]	586	585	1.04 [1.01, 1.07]	0.43 [0.21, 0.90]			0.50 [0.32, 0.78]
Park et al., 2023 COLRAR Trial [10]	151	144	1.04 [0.92, 1.17]	0.48 [0.04, 5.20]	47.00 [17.13, 76.87]		

CI—Confidence Interval; MD—Median; RCT—Randomized Controlled Trial; RR—Relative Risk; TME—Total Mesorectal Excision.

## 5. RAS: Additional Costs and Economic Efficiency

The implications of prolonged operative durations and increased material and acquisition costs in robot-assisted surgical interventions necessitate careful evaluation of their cost-effectiveness and cost-efficiency. The current evidence presents a complex landscape, highlighting both opportunities and challenges. A systematic review and Bayesian network meta-analysis of RCTs revealed that robotic surgery incurs the highest total and operative costs and the longest operative durations, despite delivering the shortest hospital stays and lowest mortality rates. However, its cost-effectiveness was found to be inferior to that of laparoscopy, though the findings were limited by the small proportion of robotic procedures included (1.65%, *n* = 70) [78]. Similarly, Singh et al. (2024) conducted a meta-analysis comparing robotic colorectal resection with laparoscopic colorectal resection, concluding that laparoscopy remains more economical in terms of operative and total costs. However, significant heterogeneity and the limited number of randomized trials necessitate cautious interpretation of these findings [79]. A retrospective single-center review of 279 elective colectomies stratified by anatomic resection provides a more nuanced perspective. The study demonstrated comparable total costs between robotic and laparoscopic colectomies overall, but identified robotic low anterior resection as significantly less costly than laparoscopic low anterior resection [80]. Notably, the implementation of a standardized robotic colectomy protocol significantly reduced operative times, operating room expenditures, length of stay, and overall costs, suggesting that standardization may improve the economic feasibility of RAS [80]. A recent scoping review further emphasized the heterogeneity in economic evaluations of RAS, with variability in cost components, methodologies, and outcomes. Critical factors, such as learning curves and shared utilization of robotic platforms across specialties, are often underexplored. High surgical volumes and structured training programs were identified as essential for improving outcomes and optimizing cost-efficiency [81].

While the cost-effectiveness of RAS remains inconclusive, targeted strategies, including standardizing protocols, leveraging robotic platforms for high-complexity procedures like low anterior resection, and considering shared use across specialties, may enhance its economic viability. Future studies should prioritize robust methodologies, including sensitivity analyses that account for procedural variability and long-term impacts, to provide clearer guidance for decision-makers.

## 6. Conclusions

RAS is increasingly applied in minimally invasive colorectal procedures and is frequently advocated for anatomically complex scenarios, such as low rectal tumors, male patients with a narrow pelvis, or obesity. However, while this rationale is widely accepted in clinical practice, these assumptions remain largely hypothetical, as stratified subgroup analyses are lacking in most available studies. 

Current evidence from meta-analyses suggests that RAS is associated with lower conversion rates. For right-sided colectomies, RAS may facilitate intracorporeal anastomosis, which is associated with improved short-term recovery in some studies and might reduce long-term complications, such as incisional hernia. In rectal cancer, recent long-term data from the REAL trial demonstrate lower local recurrence rates and improved disease-free survival for RAS compared with laparoscopy in mid and low rectal tumors, indicating a potential oncological advantage in selected patient populations. 

However, the evidence remains inconclusive. A paucity of large RCTs exists for the purpose of comparing RAS and LC in colon cancer, with the available evidence being primarily derived from large cohort studies. With regard to rectal cancer, extant meta-analyses continue to report inconsistent findings, particularly due to methodological heterogeneity, lack of patient stratification, and underreporting of surgeon- or center-related variables. Furthermore, the interpretation of economic analyses is complicated by the presence of high variability in cost structures, learning curves, and institutional setups.

Future research should prioritize subgroup analyses in RCTs focusing on high-risk populations (e.g., obesity, male pelvis, and low rectal tumors). Furthermore, there is a necessity for high-quality meta-analyses incorporating stratified data and transparent handling of heterogeneity. In addition, the performance of standardized economic evaluations that account for surgical experience, volume, and platform use across specialties is recommended in order to further investigate cost-effectiveness and cost-efficiency.

Despite the evident limitations of the available data, the available evidence suggests that RAS might be superior to LC in terms of short-term and maybe oncological outcomes. Still, procedure choice should be guided by institutional experience, patient risk profile, and resource availability while awaiting higher-quality long-term, cost, and patient-centered data.

## Figures and Tables

**Table 1:** Current evidence comparing robot-assisted and laparoscopic surgery for colon resections.

Study	Patient Focus	Design	Number of Patients	Segment Stratified	Outcomes	Long-Term Survival	Key Weaknesses
Gonçalves et al., 2024 [23]	Colon cancer	Meta-analysis, 4 RCTs	lap: 145 robotic: 148	No	Robot: ↓ LOS, ↑ wound complications	No	Underpowered; 2–3 RCTs per outcome, GRADE low certainty of evidence, no conversion data
Negruț et al., 2024 [24]	Colon cancer, recent (2020–24)	Meta-analysis, 21 studies (3 prospective cohorts, 18 retrospective cohorts)	lap: 39,712 robotic: 11,059	No	Robot: ↓ conversion, ↓ LOS, ↑ op time, ↑ lymph node yield, equivalent morbidity and positive margins	No	Lacks segmental/long-term analysis, mainly retrospective studies
Cuk et al., 2021 [25]	Colon cancer	Meta-analysis, 20 studies (16 retrospective studies, 3 prospective studies, 1 RCT)	lap: 12,059 robotic: 1740	No	Robot ↓ anastomotic leak, ↓ conversion to open (OR 0.31), ↑ op time	No	Mostly retrospective, colon/rectal sometimes mixed, 6 studies with moderate risk of bias included
Tschann et al., 2022 [26]	Right colon cancer	Meta-analysis, 25 studies (22 retrospective studies, 2 prospective studies, 1 RCT)	lap: 14,257 robotic: 1842	Yes (right only)	Robot: ↓ conversion, ↓ blood loss, ↓ LOS, ↑ op time, equivalent morbidity and oncologic outcomes	4/25 with long-term	Retrospective heavy, few with long-term, mainly retrospective studies
Zheng et al., 2022 [27]	Right colon cancer	Meta-analysis, 15 studies (12 retrospective studies, 2 prospective studies, 1 RCT)	lap: 4036 robotic: 1116	Yes (right only)	Robot: ↓ conversion (*p* = 0.03), ↓ LOS, ↑ op time; equivalent in blood loss/complications/lymph node harvest	No	Short-term metrics, no long-term follow-up, mainly retrospective studies
Morini et al., 2025 [28]	Transverse colon	Meta-analysis, 4 retrospective studies	lap: 257 robotic: 116	Yes (transverse)	Robot: ↓ LOS, ↑ op time, similar in conversion, morbidity, blood loss, time to bowel movement, lymph node yield	No	Small N, only retrospective studies (time frame 26 years)
Meyer et al., 2024 [29]	Right colon	Systematic review, 16 studies (14 cohort studies, 1 prospective study, 1 RCT)	lap: 20,200 robotic: 2489	Yes (right only)	Robot: ↓ conversion (in some studies), ↑ intracorporeal anastomosis, ↑ lymph node yield; faster bowel recovery, ↓ wound complications	One small RCT only	Mainly small/retrospective studies
Park et al., 2012 [30] Park et al., 2018 [31]	Right colon cancer	RCT (2012) + 5-year follow-up (2018)	lap: 35 robotic: 35	Yes (right only)	No significant diff. in perioperative outcomes or conversion, equivalent 5-yr DFS/OS	Yes (5 yr DFS/OS)	Sample size, single center
Sterk et al., 2023 [32]	Dutch national cancer registry, cT1–3M0 colon cancer	Retrospective cohort	lap: 14,901 robotic: 1114	Yes (right, left, sigmoid separated)	Robot: conversion ↓ in all segments (right: 4.6% vs. 8.8%; left: 4.6% vs. 11.6%; sigmoid: 1.6% vs. 5.9%); all *p* < 0.001, equivalent short-term outcomes	No	No oncologic/cost data, retrospective cohort
Dohrn et al., 2021 [33]	Right colon, Denmark national cohort	Propensity-matched cohort	lap: 718 robotic: 359	Yes (right only)	Robot: ↑ lymph node yield, ↑ intracoporal anastomosis, equivalent in morbidity and mortality	No	No oncologic data, database data
Emile et al., 2023 [34]	Colon cancer, US NCDB	Propensity-matched cohort	lap: 33,860 robotic: 6597	No	Robot: ↓ conversion, ↓ LOS, 5-yr OS: marginally better for women, generally equivalent; margin positivity similar	Yes (5 yr OS)	database data, lacks segment-level data
Cuk et al., 2023 [35]	Colon cancer, National registry of Denmark	Retrospective cohort	lap: 6905 robotic: 660	No	Robot: ↓ recurrence (robot 12.4% vs. lap 17.1%), adjusted HR for recurrence 0.7 for robot vs. lap	Yes (mean 4.9 yr)	No segment/histology data, database data
Kamel et al., 2022 [36]	T4b colon cancer, US NCDB	Propensity-matched cohort	lap: 2330 robotic: 157	No	Robot: ↓ conversion 12% vs. lap 37% (*p* < 0.001); ↓ LOS, similar OS	Yes (OS)	database data, only T4b
Tian et al., 2023 [37]	Right hemicolectomy (CME)	Multicenter, Propensity-matched cohort	lap: 223 robotic: 149	Yes (right only)	Robot: ↓ conversion, similar complications; similar 2 yr DFS/OS	Yes (2 yr)	Modest follow-up (2-yr), small N
Farah et al., 2023 [38]	Colorectal cancer, ACS-NSQIP	Propensity-matched cohort	lap: 10,950 robotic: 5475	Yes (right, left)	Robot: ↓ conversion, ↑ op time, ↑ “textbook outcome” rates, equivalent in complications/anastomotic leak/mortality	No	Database data, no long-term survival
Rein et al., 2023 [39]	Left colon, Denmark national cohort	Propensity-matched cohort	lap: 1392 robotic: 696	Yes (left only)	Robot: ↓ conversion, ↑ lymph node yield; no difference in morbidity and mortality	No	Database data, no long-term survival
Petrucciani et al., 2015 [40]	Right colon, mixed (malignant/benign)	Meta-analysis, 6 studies (5 retrospective studies, 1 RCT)	lap: 348 robotic: 168	Yes (right only)	Short-term: no sig. diff. in conversion or outcomes; patients similar	No	Malignant/benign mixed, study period 2009–2015
Lim et al., 2016 [41]	Colorectal cancer	Meta-analysis, 6 studies (4 retrospective studies, 1 prospective study, 1 RCT)	lap: 431 robotic: 253	No	Robot: shorter time to diet/flatus/defecation, ↓ LOS, ↓ blood loss, ↑ op time	No	Conversion not reported, study period 2012–2015
Solaini et al., 2022 [42]	Left colectomy only	Meta-analysis, 11 studies (11 retrospective studies, 1 prospective study)	lap: 39,083 robotic: 13,506	Yes (left only)	Robot: ↓ conversion RR 0.5 (all cases), ↑ op time, postop. outcomes similar	No	Segment only left, not subsegments, mainly retrospective studies

ACS-NSQIP American College of Surgeons National Surgical Quality Improvement Program; CME—complete mesocolic excision; DFS—disease-free survival; Lap—laparoscopic resection; LOS—length of stay; NCDB—National Cancer Database; OR—odds ratio; OS—overall survival; RCT—randomized controlled trial; yr—year; ↓ lower; ↑ higher.
